# Editorial: Electroconvulsive therapy: from mechanisms to clinical practice

**DOI:** 10.3389/fpsyt.2025.1727912

**Published:** 2025-11-10

**Authors:** Cristiana Țăpoi, Laith Alexander, Mariana Pinto da Costa, João Quevedo, Mario F. Juruena

**Affiliations:** 1 Department of Psychiatry, Prof. Dr. Alexandru Obregia Psychiatry Hospital, Bucharest, Romania; 2 Department of Psychiatry Institute of Psychiatry, King’s College London, Psychology & Neuroscience, London, United Kingdom; 3 Center for Interventional Psychiatry, Faillace Department of Psychiatry and Behavioral Sciences, McGovern Medical School, The University of Texas Health Science Center at Houston (UTHealth Houston), Houston, TX, United States

**Keywords:** electroconvulsive therapy, ECT (electroconvulsive therapy), neuromodulation, neuroplasticity, neuroinflammation, depression, catatonia, MECT

Electroconvulsive therapy (ECT) continues to be a mainstay in clinical practice due to its proven effectiveness (1–3), despite its long-standing history of stigma and controversy (4–6). In recent years, alongside technological advancements, clinical attitudes toward ECT have improved significantly (7–10). A cross-sectional survey conducted in Europe reported that early-career psychiatrists generally expressed favorable views toward ECT and a strong interest in expanding their knowledge of the procedure. Access to ECT training during psychiatry residency was associated with a greater likelihood of endorsing its safety and effectiveness, thus reinforcing the importance of continuing education and research in this area (11).

This Research Topic brings together 11 articles that cover a wide range of topics relevant to modern ECT practice. These contributions integrate insights into ECT’s mechanisms of action, clinical optimization, and the perspectives of patients, caregivers, and clinicians. By combining recent advances in neurobiological and neuroimmune research with ongoing efforts to improve clinical practice, this Research Topic highlights the dynamic bench-to-bedside trajectory of modern ECT practice.

João et al. conducted a comprehensive review of 26 studies investigating potential biomarkers related to the immune-inflammatory, structural, and cellular mechanisms underlying ECT’s therapeutic effects. Their findings provide valuable evidence of reductions in inflammatory markers, increased hippocampal neurogenesis, enhanced BDNF expression, and long-term cellular reprogramming following treatment. This helps to close the gap in our understanding of ECT’s efficacy.

Ruiz et al. presented a narrative review focusing on ECT in treatment-resistant depression (TRD). Their analysis highlights ECT’s superior efficacy in severe depression episodes, particularly life-threatening conditions where rapid symptom relief is essential, compared with other interventions known to have efficacy in TRD, such as ketamine or transcranial magnetic stimulation. The review also sheds light on the ECT’s impact on neurotransmitter systems, neurogenesis, brain networks, and the immune system, which serve as potential pathways through which ECT exerts its antidepressant effects. The authors emphasize the need for larger, longitudinal, and standardized studies to help us understand the predictors of ECT response and its underlying mechanisms.

Zhang et al. performed a retrospective analysis of the medical records of 895 patients who underwent ECT over the course of 10 months, investigating the incidence and risk factors for a fever episode within 24 hours post-treatment. The authors found that 11.6% of patients developed a fever within 24 hours after treatment, with risk factors including male sex, younger age, being treated in a closed psychiatric ward, and receiving etomidate. These results underline the importance of vigilant temperature monitoring after ECT, especially in high-risk groups.

Wang et al. conducted a retrospective study on the efficacy of ECT in patients diagnosed with schizophrenia, together with factors predicting response. Their study included 237 inpatients in China who had received ECT between January 2023 and December 2024. Their results revealed an overall ECT response rate of 70.46%. Positive predictors of treatment response included first-episode schizophrenia, higher baseline positive symptom scores, and longer EEG seizure duration, while older age and more prolonged illness duration were negative predictors. These findings emphasize the value of ECT in the early treatment of psychosis and the need for personalized ECT treatment protocols.

Bobo et al. conducted a scoping review of 82 studies evaluating ECT for perinatal depression, including its safety and efficacy. Their findings show the rapid alleviation of depressive, psychotic, and catatonic symptoms, with adverse effects typically mild and transient. However, this study also underscores the need for more research in this population, as data on fetal and neonatal safety remain limited.

Li et al. examined trends in ECT use in a psychiatric hospital in China over five years (2015-2020). Of the 22,120 inpatients admitted during this period, 10% received ECT. Emergency department referral, unstable vital signs, and severe impairment in daily functioning were independent predictors of ECT use, highlighting its role in cases requiring rapid symptom relief. This study also observed a decline in ECT utilization, from 13.2% in 2015 to 5.7% in 2020, a trend consistent with global trends. 

Patel et al. reported the case of a 35-year-old female patient with TRD and an implanted sacral neurostimulator for an overactive bladder, who safely received three sessions of ECT. At the same time, her device was placed in magnetic resonance imaging mode to prevent electrical interference. This case showed that ECT can be an effective and safe treatment option, emphasizing the importance of a thorough assessment and an individualized treatment plan in patients with neurostimulators. The case highlights the need for specific protocols for safely administering ECT in patients with implanted electronic devices.

Pinchuk et al. described two cases of patients who developed status epilepticus after ECT, which resolved through restimulation. These reports support the view that ECT has anticonvulsant properties and underscore the need to develop clear clinical protocols for restimulation in cases where conventional interventions, such as propofol or lorazepam, fail to stop prolonged seizures.

Zhou et al. explored the knowledge of, attitude toward, and willingness to undergo ECT among Chinese patients diagnosed with bipolar disorder through a questionnaire-based survey. Although knowledge levels were low and attitudes toward ECT were negative, patients expressed a surprisingly high willingness to undergo the treatment. These findings highlight the importance of clinician-led education to improve knowledge of and acceptance of ECT.

Geerts et al. conducted semi-structured clinical interviews with nine significant others of patients who received ECT to investigate caregivers’ perspectives and experiences. Participants described the emotional burden of supporting patients with severe mental illness, and said that a sense of hope accompanied the decision to commence ECT. However, they also expressed apprehension regarding potential side effects and a high sense of responsibility. Psychiatrists were identified as pivotal in fostering trust and providing accurate information. Given the crucial role of significant others in treatment decision-making and patient adherence, this study highlights the importance of clear communication from psychiatrists, efforts to counter stigma, and the provision of experience-based information to support both patients and their families.

Hosseni et al. examined the training experiences, knowledge of, and attitudes toward ECT among trainees and early-career psychiatrists in Iran via an online survey. Their findings showed a high availability of ECT centers in Iran, generally positive attitudes, and a strong interest in further ECT training. This study emphasizes the importance of exposure to and education in ECT in shaping positive professional views.

In conclusion, this Research Topic demonstrates the enduring significance of ECT in contemporary psychiatry by showcasing advances in its application while incorporating the perspectives of patients and caregivers. Together, these studies provide a comprehensive and contemporary appraisal of modern ECT practice.
